# Surveillance on speed: Being aware of infectious diseases in migrants mass accommodations - an easy and flexible toolkit for field application of syndromic surveillance, Germany, 2016 to 2017

**DOI:** 10.2807/1560-7917.ES.2018.23.40.1700430

**Published:** 2018-10-04

**Authors:** Navina Sarma, Alexander Ullrich, Hendrik Wilking, Stéphane Ghozzi, Andreas K. Lindner, Christoph Weber, Alexandra Holzer, Andreas Jansen, Klaus Stark, Sabine Vygen-Bonnet

**Affiliations:** 1 Robert Koch Institute (RKI), Berlin, Germany; 2 Department of Infectious Diseases, Vivantes Auguste Viktoria Hospital, Berlin, Germany

**Keywords:** syndromic surveillance, infection control, refugees, migrants, mass accommodation, poor living conditions, outbreaks, migrants

## Abstract

Europe received an increased number of migrants in 2015. Housing in inadequate mass accommodations (MA) made migrants prone to infectious disease outbreaks. In order to enhance awareness for infectious diseases (ID) and to detect clusters early, we developed and evaluated a syndromic surveillance system in three MA with medical centres in Berlin, Germany. Healthcare workers transferred daily data on 14 syndromes to the German public health institute (Robert Koch-Institute). Clusters of ID syndromes and single cases of outbreak-prone diseases produced a signal according to a simple aberration-detection algorithm that computes a statistical threshold above which a case count is considered unusually high. Between May 2016–April 2017, 9,364 syndromes were reported; 2,717 (29%) were ID, of those 2,017 (74%) were respiratory infections, 262 (10%) skin parasites, 181 (7%) gastrointestinal infections. The system produced 204 signals, no major outbreak was detected. The surveillance reinforced awareness for public health aspects of ID. It provided real-time data on migrants' health and stressed the burden of non-communicable diseases. The tool is available online and was evaluated as being feasible and flexible. It complements traditional notification systems. We recommend its usage especially when laboratory testing is not available and real-time data are needed.

## Background

In 2015, more than a third of newly arrived asylum seekers, refugees and irregular migrants (for the purpose of this paper, collectively referred to as newly arrived migrants) in Europe applied for asylum in Germany (n = 441,800). It is estimated, however, that the actual number that entered Germany during 2015 was twice as high (ca 890,000) [1]. The majority came from Syria, Afghanistan, Iraq, Albania, and Kosovo* [2].

On arrival in Germany, migrants were distributed among its 16 federal states and sheltered in reception centres. At that time, the existing centres lacked the capacity to support such a large number of people so mass accommodations (MA) were opened in sports halls, fairgrounds and empty buildings. However, unlike established reception centres, MA did not meet quality standards e.g. there was minimum living space per person and limited access to showers and toilets. This resulted in poor living conditions with overcrowding, poor hygiene, poor sanitation and mass catering services (without the possibility of individual food preparation); all factors that increased the risk of infectious disease (ID) transmission.

Newly arrived migrants in the EU are exposed to the same pathogens as other residents. However, there are migrant-specific factors that can affect their health such as ongoing epidemics, interrupted (public) health programmes (e.g. for primary healthcare and immunisation) in their countries of origin or in transit countries and increased vulnerability with regard to ID [3-11] due to burdensome travel (e.g. exposure to the weather, unsafe drinking water). Further, due to the large number of migrants newly arriving in Germany and the consequent administrative bottleneck there was a prolonged waiting time (sometimes several days) before accommodation and a first health check were provided.

Within the scope of the Asylum Seekers Benefits Act §§4,6 [12], asylum seekers in Germany are entitled to a limited package of healthcare including emergency medical care, treatment for acute and painful conditions, care during pregnancy and childbirth, vaccinations and other 'necessary preventive measures'. Additional care is possible if the measures are deemed to be 'essential' to preserve health [6,13].

In February 2016, the number of newly arrived migrants in Berlin peaked with more than 28,000 people living in ca 90 MA (personal communication with staff from the housing coordination centre Berlin, Regional Office for Health and Social Affairs Berlin (LAGeSo), weekly capacity update of MA, 2016). The regular healthcare system was not always accessible due to various factors such as limited capacity, uncertainty about reimbursement and lack of language and culture mediation. HCW reacted by voluntarily setting up low threshold medical centres, so called MedPoints, in various MA all over Berlin. They provided basic healthcare to migrants free of charge and guided them in how to access the regular healthcare system. MedPoints also adapted to the needs of the migrant population providing language mediation and close cooperation with social workers. In 2016, the Berlin state office for refugee affairs contracted hospitals and charity organisations to take over MedPoints in 12 large MA. Medical specialities (e.g. gynaecologists, psychologists), technical equipment and methods of data collection (computer-based, paper-based) varied significantly between MedPoints. Opening hours ranged from 2–5 days a week depending on the number of migrants in the corresponding MA. MedPoints faced two main challenges: limited resources and limited options of referral to local physicians within the regular healthcare system for further diagnostics or therapy. The latter was complicated, as migrants were required to obtain a medical treatment voucher issued by the social welfare office prior to each medical visit within the regular healthcare system.

Due to these challenges and additional care restrictions (by law), the scope of laboratory diagnostics offered to newly arrived migrants was often limited. Therefore, as the German ID notification system is mostly based on laboratory results, under-reporting of ID was likely in this population and outbreaks might not have been picked up or may have been detected late.

In 2015, the European Centre for Disease Prevention and Control (ECDC) recommended using syndromic surveillance (SySu) systems (in addition to existing notification systems) to better detect ID outbreaks earlier in migrant camps and to improve the level of information on current ID threats [5,14]. Several European countries such as Austria, France, the Former Yugoslav Republic of Macedonia, Greece and Italy had already successfully implemented such systems [15-21].

Here, we describe the development, implementation and evaluation of a communicable diseases SySu system in migrant MA in Germany. The SySu tool was based on clinical syndromes before a confirmed diagnosis, with the aim to detect ID clusters early and to enhance awareness for ID among HCW working in MA.

## Methods

### Data collection

In March 2016, we piloted a data collection sheet, which was developed together with HCW in Berlin’s largest MA (population size = 1,920 (personal communication with staff from the housing coordination centre Berlin, Regional Office for Health and Social Affairs Berlin (LAGeSo), weekly capacity update of MA, 2016)). The data collection sheet (Supplement) contained 13 syndromes or suspected ID (henceforth referred to collectively as syndromes) and a category for all non-ID. All MedPoints received syndrome definitions (Table 1). It was optional to collect data for all age groups together or split into two age categories (< 15 years and ≥ 15 years). The system (data collection sheet, mode of data transfer and outbreak-detection algorithm) was readjusted using feedback from the HCW at MedPoints during field visits.

**Table 1:** Definitions of syndromes applied during syndromic surveillance in mass accommodations for newly arrived migrants, Germany, 2016–2017 (n = 13 syndromes and one category for non-infectious diseases)

Syndrome	Definition	Differential diagnosis/possible aetiology
1. Acute respiratory infection/influenza-like illness	At least one of the following symptoms: sore throat cough rhinitis headache and/or body aches with or without fever and malaise	Influenza, especially if upsurge of cases during influenza season Pharyngitis, rhinitis, tonsillitis, sinusitis caused by other viruses or less often bacteria
2. Chronic cough ( > 3 weeks)	Cough lasting for more than 3 weeks	Pulmonary tuberculosis Whooping cough
3. Suspected pneumonia/bronchitis	Clinical signs of pneumonia or bronchitis	Viral infections Bacteria such as *Streptococcus pneumoniae, Mycoplasma pneumoniae* Legionellosis should be considered if cases are clustering or do not respond to standard antibiotic therapy
4. Suspected varicella	Diffuse generalised maculopapularvesicular rash on skin or mucus membranes, often accompanied by fever OR Other symptoms with strong clinical suspicion of varicella	Varicella
5. Suspected measles	Generalised maculopapular rash lasting for more than 3 days AND fever ( ≥ 38 °C) AND at least one of the following: cough coryza Koplik spots conjunctivitis	Measles
6. Fever with rash (no varicella, no measles)	Fever ≥ 38 °C AND skin rash	Rubella, exanthema subitum, erythema infectiosum, enterovirus infection, chikungunya-, zika-, dengue-, West Nile virus infection associated with non-specific viral exanthema, bacterial infection such as scarlet fever, typhoid fever, louse-borne relapsing fever, leptospirosis, rickettsiosis
7. Meningitis or encephalitis like syndrome	Temperature ≥ 38 °C and at least one of the following symptoms: severe headache neck stiffness altered consciousness or mental status	Meningococcal, pneumococcal or *Haemophilus influenzae* meningitis (particularly when vaccinations are incomplete), meningitis due to *Listeria* spp.*, Leptospira* spp.*,* enterovirus, West Nile virus, herpes simplex virus, tuberculosis, syphilis
8. Suspected scabies/lice	Skin lesions caused by scratching, and/or papules, vesicles, pustules, small linear burrow tracks, presence of parasites	Scabies Lice (head lice, clothes lice)
9. Vomiting and/or diarrhoea	At least three loose stools per day, and/or vomiting	Projectile vomiting for example due to *norovirus* Watery diarrhea for example due to noro-, adeno-, astro-, rotavirus, *Salmonella* spp.*, Shigella spp., Escherichia coli, Yersiniae spp., Giardia spp., Campylobacter spp., Cryptosporidium spp.* Fatigue, jaundice, enlarged liver for example due to hepatitis A virus infection Flaccid paresis (for example due to poliomyelitis) Other possibly causes (toxins or chemicals)
10. Bloody diarrhoea	At least 3 loose stools per day AND red blood in stool with or without vomiting or abdominal pain	Enteroinvasive bacterial or parasitic infections such as enterohaemorrhagic *E. coli, Clostridium difficile spp., Campylobacter spp., Shigella spp., Amoeba*
11. Jaundice of acute onset	Acute onset of jaundice and at least one of the following symptoms: fever ( ≥ 38 °C) hepatomegaly malaise	Viral hepatitis (hepatitis A), leptospirosis, yellow fever
12. Unknown/ undiagnosed/ unexplained severe infection or death	Severe disease or death of unknown aetiology, most likely caused by an infection	Fever and provenance from a malaria endemic country with cerebral symptoms and/or multi-organ failure (e.g. cerebral malaria) Sepsis or septic shock (e.g. caused by louse-borne relapsing fever, invasive meningococcal disease) Fever ( ≥ 38 °C) and swollen lymph nodes (e.g. tularaemia, diphtheria) Acute flaccid paralysis (e.g. poliomyelitis, botulism) Fever ( ≥ 38 °C) and bleeding (e.g. viral haemorrhagic fevers)
13. Other suspected infections	All infectious diseases that cannot be allocated to category 1–12	Scarlet fever, vesicular stomatitis, impetigo, urinary tract infection, visceral leishmaniasis, malaria
14. Other disease (non-infectious disease)	All other diseases that are most likely not caused by an infection	NA

NA: not applicable.

In total, four MedPoints in three MA in Berlin voluntarily participated in the project. The HCW at each MedPoint were asked to complete the data collection sheet on a daily basis and it was possible to report multiple syndromes per patient (Table 1). The aggregated numbers were reported by telephone, fax or online within 24 hours to the surveillance team at the German national public health institute (Robert Koch-Institute (RKI)) for data analysis. The algorithm generated a signal in case of unusual events: A signal was defined as an ‘unexpected high number of cases on a given day’. This is equivalent to defining a threshold, above which a daily count generates a signal. We chose a simple and intuitive, but flexible method.

The threshold *T* for a given syndrome at a given day was defined as the average *A* of daily relative-count (proportionate morbidity), defined as the count divided by the sum of counts for all syndromes on the same day, plus *n* standard deviations *S*, where *A* and *S* were computed over a preceding period *d* days: *T* = *A*(*d*) + *n S*(*d*). The default values for *n* and *d* were 2 and 21 days respectively. Based on visual inspection this seemed to generate the most relevant signals, as well as provide a sample size large enough for meaningful statistics over a period of time that was short enough to account for the changing population of the MedPoints. As some MedPoints were closed during the weekends or operated just 2 days a week, some days had no counts (number of times a given syndrome was reported on a given day). Therefore, the quantities were computed over less than *d* data points, typically 15 days for the default *d* = 21 (for MedPoints working 5 days a week). The values of these parameters could be set in order to optimise the trade-off between sensitivity and specificity, depending on the symptoms and the experience of the HCW. For acute respiratory infections and ‘other non-communicable diseases’ we set the value to n = 4; *n* and *d* were set to the standard values for the other syndromes.

**Figure 1:**
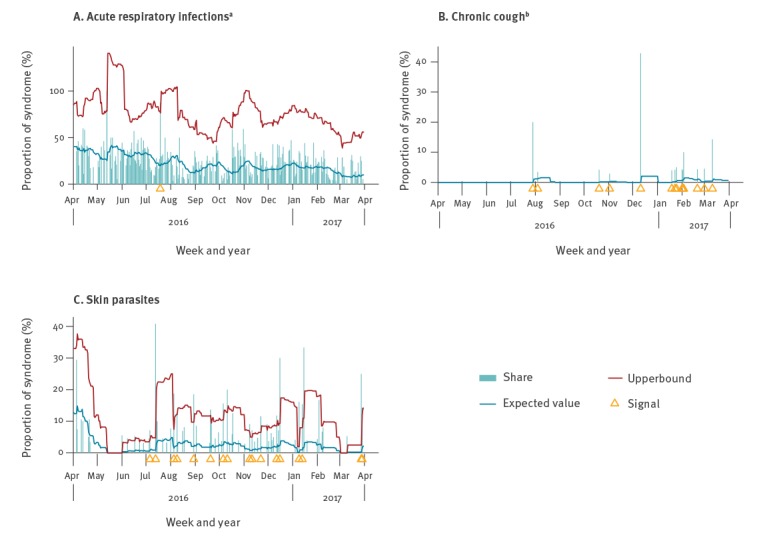
Syndromic surveillance in mass accommodations for newly arrived migrants showing signals caused by reported syndromes: (A) acute respiratory infections, (B) chronic cough and (C) skin parasites in one medical healthcare centre, Berlin, Germany, week 18/2016–week 17/2017

^a^ For respiratory infections we set the minimum number of cases for a signal to 4.

^b^ For chronic cough, a fixed threshold of 1 was chosen.

Two additional parameters allowed for further flexibility. First, the minimum daily count needed for a signal to be generated and second, a fixed threshold above which signals were always generated. They were respectively set to 1 and infinity by default. For instance, a low number of acute respiratory infections might be statistically but not epidemiologically relevant, so that only a daily count strictly above three cases could generate a signal. On the other hand, for some syndromes a single case was considered remarkable enough that it always triggered a signal; this was the case for chronic cough, varicella, measles, fever with rash, meningitis, bloody diarrhoea, jaundice, death or severe infection. Examples of count time series, thresholds, and signals are shown in Figure 1. These analysis and visualisations were carried out once the data were entered in an Excel document. Signals were visualised inside the Excel sheet as red marks [22]. All unusual syndrome clusters or single events with potentially high transmission risk were immediately communicated to the MedPoints for outbreak verification.

The y-axis shows the proportion (%) of a specific syndrome relative to all syndromes.

Our SySu toolkit is available at [22]. It consists of: (i) a data collection sheet, (ii) a Microsoft Excel (Microsoft Corporation, Redmond, Washington) file for cluster analysis consisting of sheets for data entry, indication of signals, plots, tables and a sheet for setting parameters and, (iii) supporting material for public health management of ID. The tool allows users to enter, store, analyse, visualise and re-use data and signals. This facilitates its adoption, particularly by non-scientific staff.

### Evaluation

In order to understand the SySu data in the context of the regular German notification data, we analysed the number of laboratory confirmed or clinically diagnosed outbreak cases among migrants in mass accommodations in Berlin between week 40/2015—week 13/2017, which were notified to the national notification system and compared those to our SySu data.

Moreover, after a 12 months surveillance period, we assessed the effectiveness of and experience with the surveillance system following the United States (US) Centers for Disease Control and Prevention (CDC) guidelines [23]. To assess effectiveness, we retrospectively analysed the data for completeness (quota of total number of reports received) and timeliness (reporting within 24 hours).

In addition, we asked MedPoints in April 2017 to respond to a standardised online questionnaire with 21 questions on representativeness and usefulness of the system (flexibility, feasibility and impact).

Regular meetings with all MedPoints in Berlin enabled continuous feedback of HCW on the implementation of the SySu system and information exchange between MedPoints and the RKI.

## Results

### SySu data

From 1 May 2016–30 April 2017 four MedPoints from three MA participated. The average population under surveillance was 2,109 (range 1,395–2,826). We received 810 completed documentation sheets; 9,364 syndromes were reported, of these 2,717 (29%) were ID syndromes. The majority of ID syndromes were acute respiratory infections (n= 2,017: 74%), followed by skin parasites (n = 262: 10%), gastrointestinal infections (n = 181: 7%) varicella (n = 26: 1 %) and measles (n = 3: 0.1%) (Table 2). A monthly report was disseminated to participating MedPoints and local health authorities.

**Table 2:** Numbers, incidences of cases per week per 1,000 persons (minimum, maximum, mean incidence), proportions (%) and signals of all syndromes recorded by the syndromic surveillance in mass accommodations, Berlin, Germany, week 18/2016–week 17/2017

	Total mass accommodation 1				Total mass accommodation 2				Total mass accommodation 3				Total all mass accommodations			
Syndrome/ suspected disease	Number of cases	%	Mean incidence/ 1,000 migrants (range)	Signals	Number of cases	%	Mean incidence/ 1,000 migrants (range)	Signals	Number of cases	%	Mean incidence/ 1,000 migrants (range)	Signals	Number of cases	%	Mean incidence/ 1,000 migrants (range)	Signals
1. Acute respiratory infection/influenza-like illness	1,334	21.7	25.2 (7.1‒ 48.5)	1	653	24.2	19.7 (0.0‒53.4)	4	30	5.7	1.3 (0.0‒21.9)	1	2,017	21.5	19.2 (3.5-41.3)	6
2. Chronic cough (> 3 weeks)	23	0.4	0.6 (0.0-7.9)	15	7	0.3	0.2 (0.0-4.2)	5	3	0.6	0.1 (0.0-4.4)	2	33	0.4	0.4 (0.0-2.9)	22
3. Suspected pneumonia/bronchitis	17	0.3	0.4 (0.0-4.0)	4	10	0.4	0.3 (0.0-3.2)	2	20	3.8	0.9 (0.0-13.4)	4	47	0.5	0.5 (0.0-3.7)	10
4. Suspected varicella	22	0.4	0.4 (0.0-5.2)	11	3	0.1	0.1 (0.0-4.3)	1	1	0.2	0.1 (0.0-2.6)	0	26	0.3	0.3 (0.0-2.7)	12
5. Suspected measles	3	0	0.1 (0.0-1.1)	3	0	0	NA	0	0	0	NA	0	3	0.0	0.0 (0.0-0.5)	3
6. Fever with rash (no varicella, no measles)	5	0.1	0.2 (0.0-5.8)	4	9	0.3	0.3 (0.0-3.3)	8	0	0	NA	0	14	0.1	0.2 (0.0-2.0)	12
7. Meningitis or encephalitis-like syndrome	3	0	0.1 (0.0-1.7)	3	0	0	NA	0	0	0	NA	0	3	0.0	0.0 (0.0-0.6)	3
8. Suspected scabies/lice	154	2.5	3.0 (0.0-23.8)	18	76	2.8	2.2 (0.0-16.0)	11	32	6.1	1.5 (0.0-22.6)	2	262	2.8	2.7 (0.0-14.5)	31
9. Vomiting and/or diarrhoea	91	1.5	1.8 (0.0-7.4)	13	77	2.9	2.4 (0.0-11.9)	12	9	1.7	0.4 (0.0-8.9)	2	177	1.9	1.7 (0.0-5.6)	27
10. Bloody diarrhoea	3	0	0.1 (0.0-2.0)	3	0	0	NA	0	1	0.2	0.0 (0.0-2.3)	1	4	0.0	0.1 (0.0-0.6)	4
11. Jaundice of acute onset	1	0	0.0 (0.0-0.9)	1	0	0	NA	0	0	0	NA	0	1	0.0	0.0 (0.0-0.5)	1
12. Unknown/ undiagnosed/ unexplained severe infection or death	1	0	0.0 (0.0-1.2)	1	0	0	NA	0	0	0	NA	0	1	0.0	0.0 (0.0-0.5)	1
13. Other suspected infections	85	1.4	1.5 (0.0-6.0)	67	39	1.5	1.2 (0.0-6.8)	2	5	1	0.2 (0.0-5.1)	3	129	1.4	1.2 (0.0-3.5)	72
14. Other disease (non-infectious)	4,418	71.7	90.6 (9.6-166.4)	0	1,807	67.4	56.4 (0.0-112.5)	0	422	80.7	19.8 (0.0-80.3)	0	6,647	71.0	62.5 (5.3-102.8)	0
**Total**	**6,160**	**NA**	**NA**	**144**	**2,681**	**NA**	**NA**	**45**	**523**	**NA**	**NA**	**15**	**9,364**	**NA**	**NA**	**204**

NA: not available.

**Figure 2:**
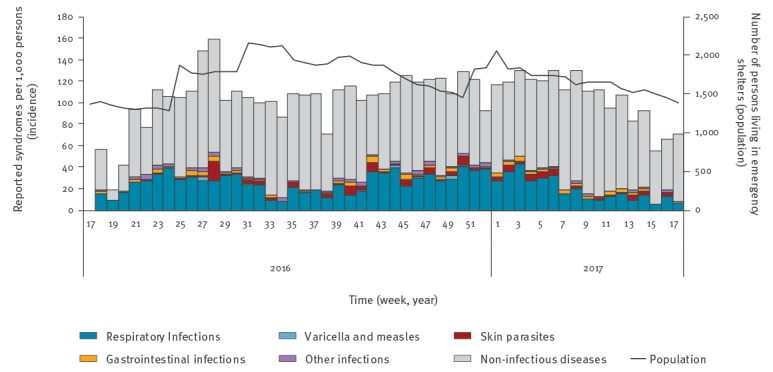
Incidence rates of all syndromes reported by medical healthcare centres, syndromic surveillance in three mass accommodations, Berlin Germany, week 18/2016–week 17/2017 (n = 9,364)

Incidence by calendar week was highest for non-ID, followed by acute respiratory infections (Figure 2). Median incidence of acute respiratory infections was 24.2 per 1,000 persons with preponderance in autumn and winter. Incidence of skin parasites and gastrointestinal infections varied considerably. Incidence of measles and varicella was low.

The bars represent the cases per 1,000 persons per calendar week recorded by all participating medical healthcare centres, stratified by disease groups; corresponding y-axis on the left of the diagram (n = 0–180). Black line represents the weekly number of all migrant housed in the respective mass accommodations; corresponding y-axis on the right of the diagram (n = 0–2500).

Respiratory infections include ‘acute respiratory infection’, ‘chronic cough’ and ‘suspected pneumonia or bronchitis’.

Gastrointestinal infections include ‘vomiting/diarrhoea’, ‘bloody diarrhoea’ and ‘jaundice’.

The daily count of acute respiratory infections (relative to all syndromes) moved below the threshold throughout the surveillance period and signals were rare (Figure 1). Skin parasites were prevalent throughout the surveillance period, in all MA, with many signals clustering on consecutive days. Overall, 204 signals were generated (38 respiratory infections, 32 gastrointestinal infections, 31 skin parasites, three suspected measles, 12 suspected varicella and 88 others) (Table 2); all signals were verified with the respective MedPoint. No signals were interpreted as an outbreak, reflecting low specificity of the system. It was not possible to evaluate the sensitivity of the system, as no outbreaks were detected by any other means.

### Evaluation

**Figure 3:**
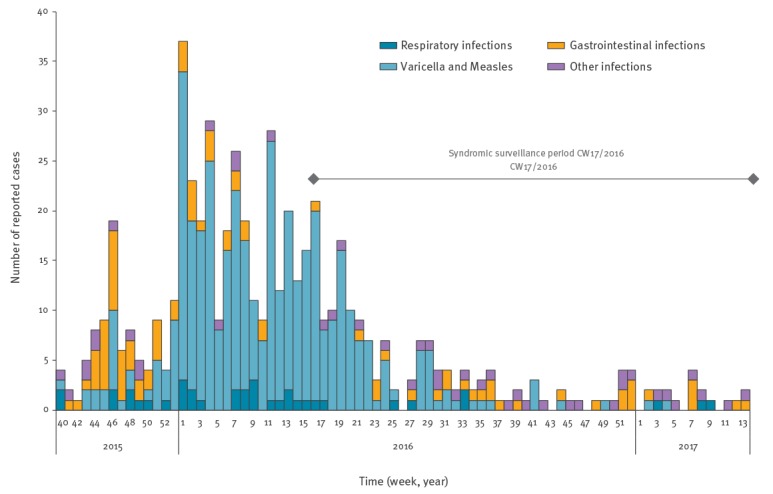
Number of laboratory confirmed or clinically diagnosed notified outbreak cases in mass accommodations, Berlin, Germany, week 40/2015-week 13/2017

Notification data show a decline of ID and outbreaks among newly arrived migrants in 2016 (Figure 3). High numbers of varicella (n = 325) and three measles cases were reported during the first 6 months of the year. Several gastrointestinal infections occurred in the beginning of the year. During the SySu period, starting in the third quarter of the year, the number of notified ID dropped, which is also reflected in our data.

Data source: SurvNet (database of the German notification system at the Robert Koch Institute).

The figure is based on the method for identification of asylum seekers in German notification data described by Kuehne et al. 2016 [24].

Analysis showed that data transmitted from MedPoints via telephone was 100% complete and delivered in a timely fashion within 24 hours. Comparatively, on average, reports sent online or by fax were delivered on time 89% and 74%, respectively.

We received nine evaluation sheets from HCW from the four participating MedPoints. HCW were asked to estimate the proportion of all inhabitants in MA that preferred to use the regular healthcare system rather than services offered by the MedPoints. Their answers ranged from 10% to 90% (total numbers are not known). Three out of nine participating HCW stated that MedPoints were informed by clinics and general practitioners when patients went to seek healthcare in the regular healthcare system and infectious diseases were diagnosed. Seven estimated that the SySu data reflected the ID situation in the respective MA.

HCW at all four MedPoints confirmed that the system was capable of adapting to the varying conditions between the different MedPoints and MA in a flexible manner. Eight out of nine HCW stated that case definitions were clear and instructions were easy to follow. To complete the surveillance form, HCW required 5–15 min/day. More than half of the participating HCW (n = 5/9) thought that it would be feasible to continue the surveillance on their own if materials were provided.

Of nine HCW, three thought the surveillance was useful, one thought the surveillance triggered changes in MedPoints, such as optimisation of ID management, and five HCW stated that attention to ID control was improved by the implementation of the surveillance. In one MedPoint, the surveillance system triggered the development of an outbreak investigation quality management and aided in the improvement of communication with local health authorities.

During regular meetings with HCW, coordinators and language mediators of all MedPoints it was found that ID were of relatively low priority. The most challenging issues were chronic illness, mental health and child well-being. MedPoints faced considerable difficulties to adequately respond to those needs. Furthermore, the meetings turned out to be an important space for information exchange between different MedPoints.

## Discussion

In 2015, many newly arrived migrants in Germany were housed in provisionally arranged MA with poor living conditions. This and an increasing number of reports of ID outbreaks among migrants in community shelters in 2014 [24], required a quick public health response. Consequently, the RKI developed an online SySu tool to augment the regular notification system with timely surveillance information. It was designed for flexible and rapid use in MA with integrated medical services and allowed for easy adaptation of syndromic categories and parameters. We based the SySu system on examples and lessons learned from other European Union countries and adapted it to the specific situation of migrants arriving in Germany, who had already transited through several other countries. Integrating HCW’s expertise from MedPoints in Berlin during the whole process was essential.

In 2016, the system documented the absence of outbreaks in three of the largest MA in Berlin. This is in line with observations of other European SySu systems [15,16,18,20] and the German notification data during our surveillance period. We observed an improvement of public health interventions including vaccination, health information and increased hygiene with the progressive professionalisation of MedPoints. For example, in January 2016, varicella and measles outbreaks were controlled by targeted vaccination programs [25]. The mandatory notification data did not show the high prevalence of acute respiratory infections and skin parasites, which we observed in our SySu data and which was also reported by other SySu examples, e.g. Italy [20]. Moreover, the SySu provided information on the share of ID and non-ID syndromes. This confirms the added value of SySu data in providing information on the global disease burden and not just being limited to notifiable diseases.

### Limitations

Specificity of the detection algorithm was low, a known limitation in SySu systems [23,26-30]. In the long term, parameters should be set as to optimise the balance between sensitivity and specificity. Moreover, the actual proportion of the migrant population in MA covered by the surveillance was not known due to incomplete information on the proportion of persons seeking medical help elsewhere. In addition, the population was highly mobile and information on the actual number of people coming and going to the MA was unavailable. Therefore, disease conditions of the population already residing in MA and that of newly arriving individuals could not be disentangled.

Further, a list of all MedPoints operating in Berlin was initially not available and it took several weeks until we could invite all MedPoints to participate. Due to high workload, limited resources, lacking operation standards and the fact that HCW questioned the benefit of the surveillance, the overall rate of participation was low. During regular meetings with MedPoint personnel we learned, that ID, while present in high numbers, were not the major health concerns in MA. HCW were challenged by an increase in psychological disorders, which is a known effect of continued stay in migrant shelters [29]; our system did not allow for detailed data collection on those conditions. Another crucial problem for MedPoints was the struggle of integrating newly arrived migrants into the regular healthcare system and to ensure regular developmental check-ups for children and vaccination for infants. In most cases, the contracts for the MedPoints did not include extra capacity for those tasks.

Regular personal contact between the RKI team and HCW proved to be essential for data quality. While data transmission through telephone proved to be timely and complete, online and fax transfer were not as efficient. Data transmission by telephone was effective on a regional level, where the set-up is more confined but might not be possible on a larger/national level. Response to the evaluation questionnaire was low so the informative value of its results is limited; the evaluation does not provide an accurate assessment of representativeness. Nevertheless, the SySu system proved to be feasible in all four MedPoints and provided a positive impact. The surveillance system raised awareness for ID and outbreak management among HCW and started continuous exchange of ideas and good practices (e.g. for performance of laboratory diagnostics despite of not being included in the budget) among MedPoints, which further contributed to closer cooperation with local health authorities. Moreover, demands to decision makers could be collectively communicated, leading to some improvements e.g. more paediatric consultation hours were budgeted. Furthermore, it provided an opportunity to the RKI to get an insight into the field situation and a better understanding of the health situation in MA and the work of MedPoints. The surveillance tool also enabled easy production of consultation statistics for internal documentation.

## Conclusion

We found that a SySu tool can be readily implemented in MA with integrated MedPoints. The tool was easy to use, could be flexibly adapted to variable conditions in MA and applied in similar settings to better document the disease burden among migrant populations. We recommend using SySu not only for early outbreak detection, but also to raise awareness of ID among frontline HCW, in order to collect real time data that is not included in the national surveillance and that can be acted upon quickly if an outbreak situation occurs. To be effective, we recommend regular communication among all stakeholders and the initiation of a network among MedPoints to exchange good practices. In 2015, the migrant situation in Europe triggered the development of expertise concerning ID management in migrant settings, including Germany. In October 2016, the ECDC published a handbook on implementing SySu in migrant reception/detention centres and other migrant settings [26].

The newly formed expertise should lead to better preparation for similar future situations in Europe.

## Note

*This designation is without prejudice to positions on status, and is in line with United Nations Security Council Resolution 1244/99 and the International Court of Justice Opinion on the Kosovo declaration of independence.
